# Modification of RECIST 1.1 criteria for assessing response in breast tumours treated with radiation therapy using multiparametric breast MRI: Radiology and oncology perspective

**DOI:** 10.1016/j.breast.2026.104750

**Published:** 2026-03-12

**Authors:** E. Durie, M. Morris, N.A. Healy, S. Salehi-Bird, A. Seth, P. Haria, N. Tunariu, S. Allen, Aditi Chandra, Shalini Sahu, Rakesh Anandarajan, Miklos Barta, Ioannis Roxanis, F.H. Cafferty, M.D. Blackledge, N. Somaiah, the KORTUC phase II trial management group

**Affiliations:** a Division of Radiotherapy and Imaging, The Institute of Cancer Research, Sutton, UK; b The Royal Marsden NHS Foundation Trust, Sutton, UK; c Department of Bioengineering, Imperial College London, UK; d Department of Radiology, Royal College of Surgeons in Ireland, Dublin, Ireland; e Beaumont Breast Centre, Beaumont Hospital, Dublin, Ireland; f Cambridge Breast Unit, Cambridge University Hospitals NHS Foundation Trust, Cambridge, UK; g University Hospitals North Midlands, Stoke, UK; h NHS Greater Glasgow and Clyde, Glasgow, UK; i Tata Memorial Centre, Mumbai, India; j Tata Medical Centre, Kolkata, India; k Christian Medical College, Vellore, India; l Regional Cancer Centre, Thiruvananthapuram, India; m Royal Cornwall Hospitals NHS Trust, Truro, UK; n The Breast Cancer Now Toby Robins Research Centre, The Institute of Cancer Research, London, UK; o Clinical Trials and Statistics Unit, The Institute of Cancer Research, Sutton, UK

**Keywords:** Radiation therapy, Primary breast radiotherapy, MRI response assessment, Multiparametric MRI

## Abstract

The traditional, size-based, RECIST 1.1 guideline is the standard for assessing tumour response, but has notable limitations, particularly for breast tumours treated with radiotherapy (RT). RT often induces fibrosis, leading to a persisting measurable abnormality on T1W/T2W MRI sequences despite an underlying pathological response. As pre-operative RT trials expand and omission of surgery is being tested, having anatomico-functional MRI response criteria may facilitate more accurate response evaluation. We propose modified RECIST 1.1 criteria based on 3 sequences of multiparametric MRI (mpMRI): unenhanced T2W, contrast-enhanced (CE) T1W and diffusion-weighted imaging (DWI). Key recommendations include complete response defined as 1) resolution of tumour mass on all sequences, or 2) evidence of residual T2 abnormality with CE images showing no enhancement above background parenchymal enhancement (BPE) and DWI signal consistent with necrosis/fibrosis, or 3) minimal evidence of enhancement above BPE with DWI in keeping with fibrosis. Criteria are also defined for other response categories (partial response/stable disease/progression). These recommendations can be tested in future trials and offer guidance on the interpretation of mpMRI sequences when breast tumours are treated in situ with radiation, a scenario in which little published data currently exists. They highlight how DWI can aid RT response assessment and help overcome some limitations with current RECIST criteria.

## Highlights

• Size-based RECIST 1.1 criteria have notable limitations.
• Breast tumours develop fibrosis when treated with primary RT.
• Multiparametric MRI images can help distinguish fibrosis and residual disease.
• Modified criteria for RT response assessment with multiparametric MRI are presented.

## 1 Introduction

Contrast-enhanced magnetic resonance imaging (CE-MRI) of the breast is the ‘gold standard’ modality to assess pre-operative (neoadjuvant) treatment response in breast cancers (BCs) [1]. Commonly patients with BC are treated with chemotherapy with or without immunotherapy/biological therapies in the pre-operative setting, with RT typically delivered post-surgery in the adjuvant setting. Numerous studies have evaluated the response of BCs to pre-operative chemotherapy using MRI, correlating MRI findings with histopathological responses post-surgery [[2], [3], [4]]. CE-MRI has proven to be a valuable tool for identifying early responders and predicting pathological complete response (pCR) [5].

The most widely used oncological response criteria, RECIST 1.1 describe responses based on changes in longest dimensions of measurable lesions, without incorporating functional MRI parameter changes that may occur in response to treatment [1,6]. RECIST criteria are imaging modality agnostic and although they recommend use of contrast (where applicable) they lack guidance on how to interpret changes in enhancement. There have been several reports of low reliability of RECIST 1.1, as it may miss clinically meaningful responses where there is not a marked tumour size reduction [7]. This is particularly relevant for patients treated with RT as a T2-weighted abnormality often persists, with RT-induced fibrosis within the tumour region and surrounding stroma.

## 2 Indications for primary RT in breast cancer

RT is predominantly used in the post-operative setting. However, with advances in systemic therapies and prolonged life expectancies, RT is increasingly employed for long-term loco-regional control when surgery is not the preferred option, e.g. presence of metastatic disease, significant comorbidities, or patient preference [8]. In recent years, there is also significant growing interest in pre-operative RT for early BC, leveraging immune-modulatory effects of RT, which eventually may enable surgical de-escalation [[9], [10], [11], [12]]. Consequently, accurately defining and assessing RT response on MRI is vital.

## 3 Studies evaluating MRI response to primary breast cancer RT

There is limited data on the use of MRI to evaluate response to RT of breast tumours in-situ. A review looking at MRI response after pre-operative RT identified 5 relevant studies including 176 patients [13]. One study treated patients with pre-operative accelerated partial-breast RT, comparing histopathological response and MRI response using RECIST 1.1. Of 48 patients, 8 demonstrated CR on MRI, with 7 of these achieving (nearly) pCR (PPV = 87.5%). However, among the 40 patients classified as incomplete responders on MRI, 6 (15%) actually achieved (nearly) pCR, representing false negatives [14]. Another study testing low dose pre-operative RT and chemotherapy found RECIST 1.1 response on MRI to have good sensitivity but poor specificity at predicting responders on histopathology, attributing this to MRI's limited capability of differentiating scar tissue from residual tumour [15]. Other small studies with single ablative dose pre-operative RT found MRI to have moderate correlation with pCR [16]. Clearly further studies are required to investigate radiation response on MRI, and improvements can be made in the capability of MRI to predict response by incorporating functional MRI sequences.

## 4 Challenges in assessing RT response on MRI

Distinguishing between low volume residual disease and post-treatment fibrosis/scarring with complete response (CR) is a major challenge. Multiple studies in the pre-operative chemotherapy setting have reported cases where patients with incomplete imaging responses were found to have pCR [17,18]. In these cases, fibrous granulation tissue, rich in small vessels and granulation structures, likely caused subtle late-phase enhancement, mimicking residual tumour tissue [19,20]. Conversely, MRI can also underestimate disease extent.This is particularly problematic with invasive lobular cancers, which can present as non-mass lesions, complicating interpretation of imaging [21].

In the pre-operative or primary RT setting fibrosis is a common problem as seen in rectal cancers, where a specific MRI-based tumour regression grading system (mrTRG) considering level of fibrosis, has been developed to assess response [22]. Accurate response assessment in this setting has become paramount with a move towards ‘watch-and-wait’ surveillance in the clinical complete-responders [23]. mrTRG cannot be directly applied to breast tumours due to differences in tissue composition, anatomy and typically low T2 signal of breast tumours, but highlights that differing response criteria are often required in the post-RT setting.

## 5 MRI sequences required for RT response assessment

For accurate response assessment the same acquisition protocol should ideally be followed at each timepoint for each individual patient [24]. A standard acquisition protocol should include standard T2-and T1-weighted CE images. Subtraction images (created by subtracting a pre-contrast image from a post-contrast image at a certain timepoint [25]) are widely used in clinical practice and felt to be crucial for accurate response assessment. Subtraction images should be generated at a minimum of ∼70s, ∼140s, and ∼450s to facilitate quicker and more consistent radiologist review. Given the high fat content of breast tissue, fat-suppressed images are essential.

Diffusion-weighted MRI (DW-MRI) is widely used in oncological imaging with numerous papers reporting correlation between degree of lesion cellularity and presence of necrosis on histopathology, and changes observed in apparent diffusion coefficient (ADC), the DWI quantitative parameter [26,27]. The measurement typically consists of two separate acquisitions at low and high b-value and a computer-generated ADC map depicting mono-exponential decay of signal between the two acquisitions. B-value measures the degree of diffusion weighting applied; low b-values (<200s/mm^2^) are highly influenced by perfusion, whereas at higher b-values (>800s/mm^2^) the effect of capillary vasculature is lost, and persistence of high signal with low ADC correlates strongly with cellularity [28]. Therefore functional assessment derived from DW-MRI always includes interpretation of low and high b-value images and the ADC map [29]. DW-MRI is becoming more widely used in breast MRI, and ADC has proven to be useful at predicting pre-operative chemotherapy responses [3,30]. Studies from other cancers including head and neck and pancreatic cancers have shown that DWI can help distinguish between fibrosis and residual disease, a common problem in the post-RT setting [31,32]. Parameters for DWI should ideally follow EUSOBI consensus guidelines [33].

## 6 Suggested recommendations for modified MRI response criteria

Since RECIST 1.1 are the most widely used tumour response criteria, and treating with radiation typically involves a localised target lesion, we suggest a modified version of RECIST 1.1 to address the specific challenges described above.

Proposed criteria are presented in Table 1.

**Table 1:** Modified RECIST 1.1 criteria on MRI reporting.

	Response	T2	Contrast	Diffusion^a^
**1**	**Complete response**	No residual measurable mass	No residual abnormal enhancement	No measurable abnormality
**2A**		Residual T2 measurable abnormality	No enhancement above BPE in the area of residual T2w abnormality	DWI signal in keeping with necrosis or fibrosis (see Fig. 1)
**2B**		Residual T2 measurable abnormality	Minimal enhancement above BPE in the area of residual T2w abnormality	DWI signal in keeping with fibrosis (see Fig. 1)
**3**	**Partial response**	Residual measurable tumour	Contrast enhancement above BPE with tumour reduced in size by >30%	DWI signal in keeping with hypercellular disease ± necrosis (see Fig. 1)
**4**	**Stable disease**	Stable measurable tumour	Contrast enhancement above BPE with stable size of tumour (i.e. not decreased by >30% or increased by >20%)	DWI signal in keeping with hypercellular disease ± necrosis (see Fig. 1)
**5A**	**Progressive disease**	Increase in size of measurable tumour	Contrast enhancement above BPE with increased tumour size >20% OR new foci of enhancement within target lesion	DWI signal in keeping with hypercellular disease (see Fig. 1)
**5B**		New foci of tumour outside target lesion/locoregional spread	New foci of contrast enhancement outside the target lesion	DWI signal in keeping with hypercellular disease (see Fig. 1)

BPE: Background parenchymal enhancement.

a If image quality adequate.

**Fig. 1:**
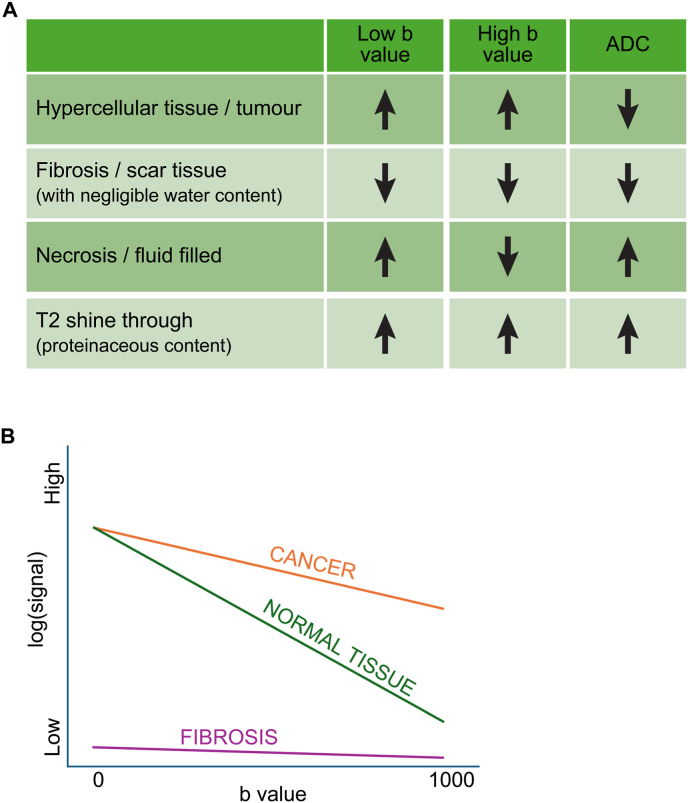
A) Schema summarising the expected signals at low and high b-value diffusion and ADC images for tumours, fibrosis, necrosis, and T2 shine-through effects. B) In the monoexponential model ADC is calculated from the slope of a best fit line between at least two b-values. Fibrosis and tumours may both show low ADC, but fibrosis has significantly lower signal at both high and low b-values.

### 6.1 Complete response (CR)

We suggest, as per RECIST 1.1, CR should be defined as the absence of any tumour mass on unenhanced T2W, CE- or DW-MRI. However, it could also be defined as no evidence of enhancement above BPE in the T2W measurable abnormality with DWI in keeping with necrosis or fibrosis. If there is late phase enhancement just above BPE, with DWI in keeping with fibrosis, CR can be assigned. Densely fibrotic tissue lacks water and therefore shows no signal at low or high b-value, and an apparent low ADC is seen in this scenario. This reflects lack of water and minimal signal decay between the two b-value acquisitions and does not indicate residual tumour (Fig. 1A and B). However, diffusion image quality must always be checked as lack of signal at low and high b-value can be due to poor quality images and may occur in tumours at the air tissue interface causing significant distortion.

### 6.2 Partial response (PR)

PR is classed as a reduction in tumour size of more than 30% (as per RECIST 1.1) with enhancement above BPE and DWI in keeping with some residual hypercellular disease ± necrosis.

### 6.3 Stable disease (SD)

SD is stable tumour size (i.e., no increase >20% and no decrease >30%) as per RECIST 1.1. DWI is likely to show evidence of hypercellular disease or minimal change.

### 6.4 Progressive disease (PD)

We suggest PD is classed as any new foci of disease, whether inside or outside the target lesion, or an increase in tumour size of >20% from its lowest recorded size (nadir). This 20% threshold aligns with RECIST 1.1 guidelines. DW-MRI is likely to show evidence of hypercellular disease in PD.

### 6.5 Disease measurement criteria

**Fig. 2:**
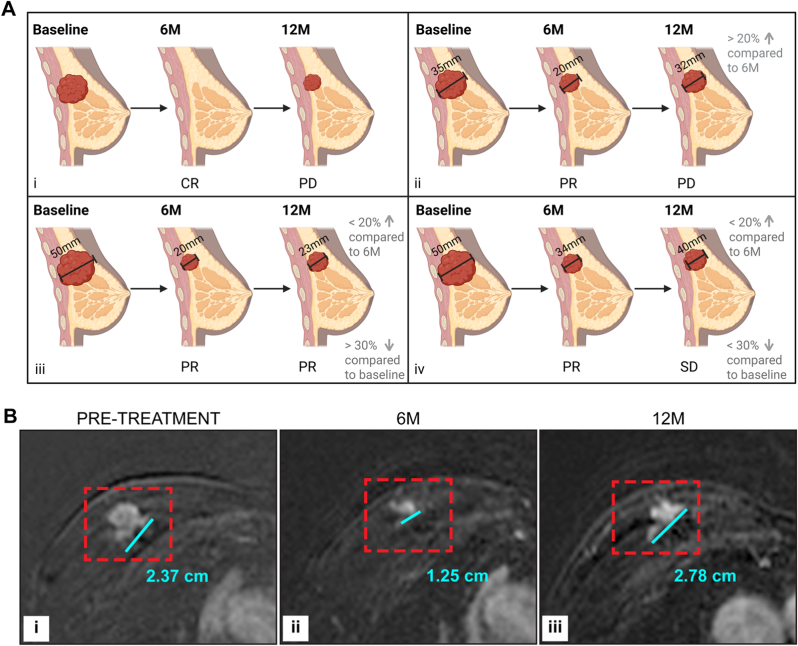
Which scan to compare with, when assessing response? A. i) Complete resolution of tumour at 6M indicating CR; at 12M tumour regrowth is seen, this is smaller than baseline but larger than the nadir (no tumour), so response is categorised as PD. ii) Tumour has shrunk by >30% at 6M indicating PR; tumour has grown by >20% at 12M compared to 6M (although remains smaller than at baseline), this indicates PD. iii) Tumour has shrunk by >30% at 6M indicating a PR; at 12M tumour has grown from 6M but by <20% and remains >30% smaller than baseline so is a continued PR. iv) Tumour at baseline has shrunk by >30% at 6M indicating a PR; at 12M tumour has grown but by <20% compared to the nadir at 6M and is now at <30% change compared to baseline so this is SD. Created in BioRender. Durie, E. (2025) https://BioRender.com/gadmjw2. B. i). Fat suppressed 70 s subtraction images showing a 2.37 cm enhancing right breast tumour (within red dashed box) at pretreatment with ii) a partial response at 6M (tumour = 1.25 cm, >30% reduction) but then iii) progressive disease at 12M (tumour = 2.79 cm). This is not >20% growth from baseline but is >20% than the nadir at 6M so is classed as progressive disease. (For interpretation of the references to colour in this figure legend, the reader is referred to the Web version of this article.)

Volumetric assessment of disease has been shown in the literature to be more accurate than change in longest tumour measurement [34,35]. However, due to clinical time constraints most centres do not use this to assess response. Another important issue is determining whether to compare against pre-treatment imaging or most recent imaging. We suggest following standard guidelines to compare to both baseline and most recent imaging. PD should be assessed against the nadir of the disease, while PR should be compared to baseline imaging (Fig. 2).

## 7 Exemplar cases

Here we highlight some cases from patients who have received high dose RT as part of the KORTUC phase II trial (NCT03946202), to illustrate breast tumour response to radiation on mpMRI, for practical application of the criteria. Although we focus mainly on tumour response, it is important to note that patients have undergone high doses of radiation to the whole breast, which can lead to significant oedema, breast shrinkage, fibrosis and skin thickening of normal breast surrounding the tumour. This is evident in many of the post-RT images included.

### 7.1 Complete responses (Fig. 3)

**Fig. 3:**
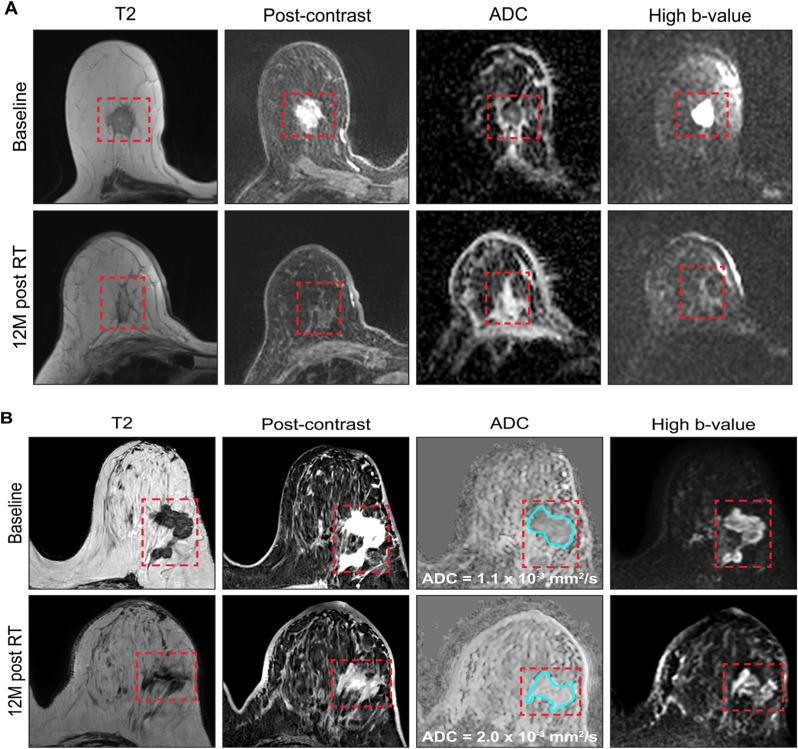

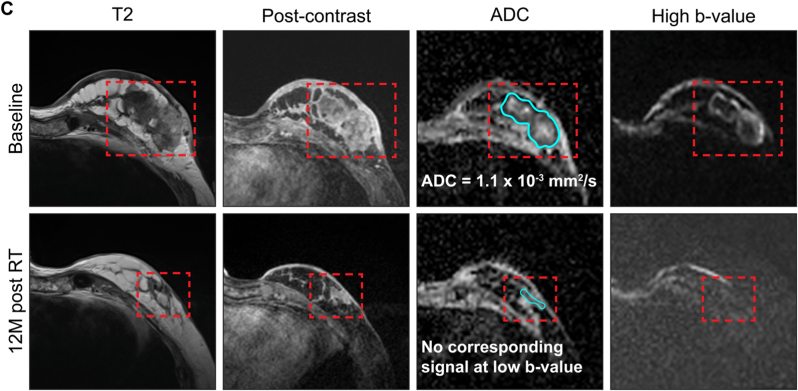
Examples of complete responses to RT at 12M post-treatment. A) CR with residual T2 abnormality (response category 2A). B) CR with T2 shine through (demonstrated by a high signal at high b-value at 12M with high ADC). C) an example of a complete response with fibrosis (demonstrated by low ADC but no corresponding signal at low or high b-value suggesting no water is present – response category 2B).

Fig. 3A demonstrates a CR with residual T2W abnormality (response category = 2A, Table 1). A well-defined enhancing mass at baseline with low ADC and high signal at high b-value within the tumour in keeping with high cellularity disease is shown. At 12M post-RT a residual T2 abnormality remains but there is no contrast uptake in this abnormality and no areas of low ADC.

Fig. 3B highlights a case of T2 shine-through. At baseline an enhancing tumour is visible with high cellularity (low ADC and high signal at high b-value). This shows a good response at 12M but some minimal enhancement, high ADC and residual high signal at high b-value, therefore indicating proteinaceous material.

A case of fibrosis is demonstrated in Fig. 3C: at baseline a large bilobar tumour is shown with some rim-enhancement, a necrotic centre and enhancement involving the skin. There is low ADC at the tumour edge with corresponding high signal at high b-value indicating hypercellular disease. At 12M there is significant tumour shrinkage, some late-phase enhancement and a small area of low ADC with no corresponding signal at low or high b-value suggesting lack of water in keeping with fibrosis (response category = 2B, Table 1).

### 7.2 Early very good PR 2-weeks post-RT (Fig. 4)

**Fig. 4:**
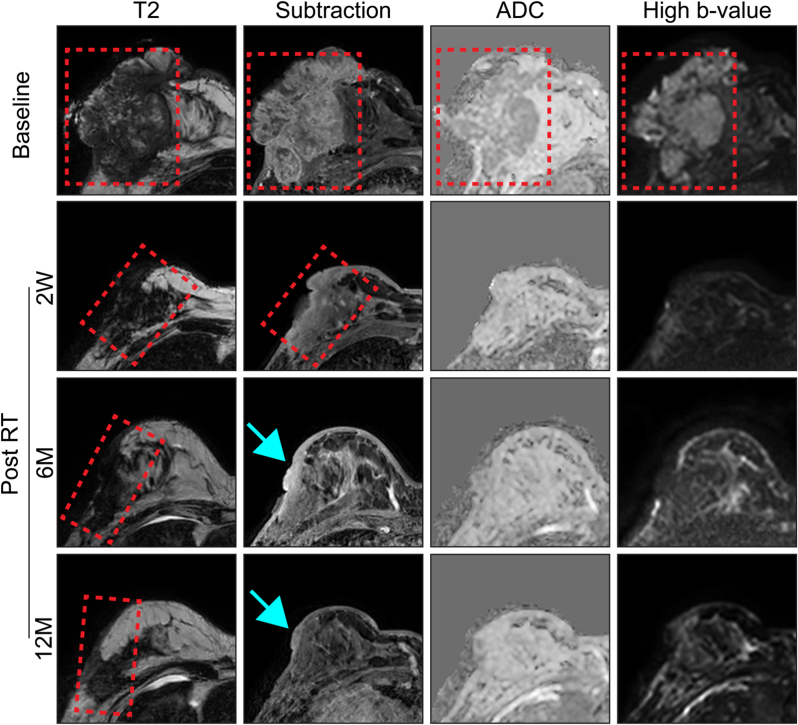
Patient with a large fungating tumour at baseline showing enhancement on subtraction images and areas with low ADC with corresponding high signal at high b-value (hypercellular disease). This patient has a very good partial response to RT at 2 weeks post-treatment; there is some enhancement in the skin, high ADC and high signal at high b-value suggesting T2 shine-through. At 6M and 12M there remains only a small area of skin enhancement (arrow), there is low ADC at 12M (red dotted box) but no corresponding signal at (low or) high b-value suggesting a CR with skin fibrosis post-RT (response category = 2B). (For interpretation of the references to colour in this figure legend, the reader is referred to the Web version of this article.)

Tumours can show very early responses to RT, as illustrated in Fig. 4, where the tumour has had a very good PR to radiation at only 2-weeks post-treatment. This becomes a CR at 6M and 12M post-RT with only a small area of enhancement in the skin, consistent with skin fibrosis.

### 7.3 PR then PD with significant RT-induced oedema (Fig. 5)

**Fig. 5:**
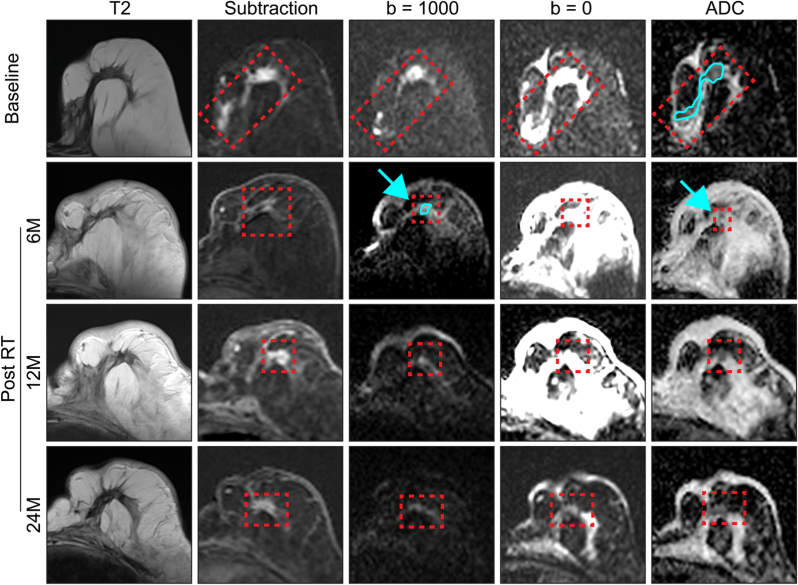
At baseline (top row) there is a clear tumour seen on the subtraction image with a bright central nodule, the tumour extends obliquely, posteriorly and medially out to the skin. The central nodule is clearly seen at high b-value and this area corresponds to the area of low ADC (hypercellular disease). At 6M post treatment there is extensive post-treatment changes and oedema post-RT shown by extensive high signal at low b-value with high ADC. A very good partial response is seen but there remains a tiny area of low ADC (highlighted by blue arrow) with corresponding area of high signal at high b-value (hypercellular disease). At 12M and 24M this area continues to slightly increase in size and enhancement showing a PD at 24M on size criteria (when compared to nadir at 6M). (For interpretation of the references to colour in this figure legend, the reader is referred to the Web version of this article.)

At baseline the tumour has a bright central nodule on CE images, with extension to the skin. A very good PR is seen at 6M with only a small area of enhancement remaining in the central nodule, corresponding to a tiny area of low ADC. This has increased in size at 12M with a larger area of enhancement and high cellularity with low ADC/high signal on high b-value image. A further increase in size is observed at 24M and it is now PD. Fig. 5 also illustrates the extensive oedema that can occur in the entire breast after high-dose RT. At low b-value there is widespread high signal with corresponding high ADC at 6M indicating the presence of large amounts of non-restricted water, this is reduced but remains high at 12M.

## 8 Testing our recommendations in a small subset (n = 12) with histopathological correlation

To evaluate the ability of our suggested criteria to predict pathological response we examined the concordance of MRI assessed response at 12- or 24–months using the revised criteria and histopathology from core breast tumour/tumour bed biopsies obtained at the same timepoint in 12 patients. The revised criteria showed concordance with histopathology in 10/12 cases (5/10 no tumour present and CR on MRI, 5/10 tumour present in biopsy and PR/PD on MRI, ability to predict CR: PPV = 100%, NPV = 71%). Of the two discordant cases, one biopsy showed atypical cells, and the histopathologist felt that the invasive tumour focus had likely been missed. This was consistent with MRI findings, which demonstrated diffuse, patchy PD. The other discordant biopsy was from the patient illustrated in Fig. 5, a small nodule remained on MRI which may have been missed on biopsy especially as these were performed under ultrasound (not MRI) guidance (and before the MRI results were available). Further larger trials are required to test the suggested criteria ideally with histopathological correlation and comparison to standard RECIST 1.1 criteria.

## 9 Important limitations of DW-MRI

DWI is emerging as a useful adjunct in breast MRI evaluation, but it is important to be aware of its limitations. Most commonly a single-shot echo planar sequence (ssEPI) is used to provide rapid imaging allowing for multiple averaging to improve signal-to-noise ratio and reduce sensitivity to motion. Imaging bilateral breasts requires a large field of view, meaning the spatial resolution is reduced with a larger slice thickness than that used in typical contrast imaging. This means very small nodules could be missed if using DWI alone. The extensive air-tissue interface in the breasts can also cause significant magnetic field inhomogeneities leading to image distortion in the region of interest and it is important to be aware of this when assessing tumours close to skin [36]. Improvements can be made to the image quality by using multi-shot echoplanar techniques or more recently deep learning reconstruction of ssEPI [37,38]. However, as advances in imaging technology are brought into clinical practice and resolution and quality of clinical DWI improves, it is poised to play an increasingly important role in evaluating response.

## 10 Conclusion

We propose a modified RECIST 1.1 criteria for breast mpMRI. It offers guidance on interpretation of unenhanced, CE and DW-MRI images and is designed to provide a more accurate assessment of response following RT for breast tumours treated in situ, an area in which there is little published literature. However, many of the principles from the guidance can be applied more broadly to other treatment settings. These proposed criteria may be incorporated in future clinical trials and will become increasingly relevant given the significant expansion of trials looking at pre-operative RT in BC [11]. Developments in quantitative mpMRI for therapy monitoring can be incorporated into these response criteria, once parameters are validated in future studies.

## CRediT authorship contribution statement

**E. Durie:** Writing – review & editing, Writing – original draft, Methodology, Data curation, Conceptualization. **M. Morris:** Writing – review & editing, Methodology, Data curation, Conceptualization. **N.A. Healy:** Writing – review & editing, Methodology, Conceptualization. **S. Salehi-Bird:** Writing – review & editing, Methodology, Conceptualization. **A. Seth:** Writing – review & editing, Methodology, Conceptualization. **P. Haria:** Writing – review & editing, Methodology, Conceptualization. **N. Tunariu:** Writing – review & editing, Methodology, Conceptualization. **S. Allen:** Writing – review & editing, Methodology, Conceptualization. **Aditi Chandra:** Writing – review & editing, Methodology, Conceptualization. **Shalini Sahu:** Writing – review & editing, Methodology. **Rakesh Anandarajan:** Writing – review & editing, Methodology, Conceptualization. **Miklos Barta:** Writing – review & editing, Methodology, Conceptualization. **Ioannis Roxanis:** Writing – review & editing, Validation. **F.H. Cafferty:** Writing – review & editing, Methodology, Data curation. **M.D. Blackledge:** Writing – review & editing, Methodology, Conceptualization. **N. Somaiah:** Writing – review & editing, Writing – original draft, Methodology, Investigation, Data curation, Conceptualization.

## Ethical approval

Ethical approval for the clinical trial linked to this study was granted by the West of Scotland Research Ethics Committee (REC reference 20/WS/0019).

## Funding

KORTUC INC. for the Phase II trial. Global Challenges Research Fund (Research England, UK). Cancer Research UK Clinical academic fellowship funding (ED). Cancer Research UK Convergence Science PhD studentship (MM). The funding bodies had no role in the study design, collection, analysis/interpretation of data or writing of the manuscript/decision to submit the manuscript for publication.

## Declaration of competing interest

The authors declare that they have no known competing financial interests or personal relationships that could have appeared to influence the work reported in this paper.
