# Supplementary material for: Modification of RECIST 1.1 criteria for assessing response in breast tumours treated with radiation therapy using multiparametric breast MRI: Radiology and oncology perspective
Source: Breast. 2026 Mar 12;87:104750. doi: 10.1016/j.breast.2026.104750 (PMC13014914; doi:10.1016/j.breast.2026.104750)
Supplement: Multimedia component 1 [file mmc1.docx]

**Appendix 1**

**KORTUC Phase II Trial Management group** (in alphabetical order):

**Navita Somaiah, Consultant Clinical Oncologist & Clinician Scientist (CI), The Institute of Cancer Research, London, UK**

Abdulla Alhasso, Consultant Clinical Oncologist (PI), Beatson Oncology Centre, Glasgow, UK

Steven Allen, Consultant Clinical Radiologist, The Royal Marsden NHS Foundation Trust, London, UK

Carmel Anandadas, Consultant Clinical Oncologist (PI), The Christie NHS Foundation Trust, Manchester, UK

Selva Anbalagan, HSO, The Institute of Cancer Research, London, UK

Selvamani Backianathan, Consultant Clinical Oncologist (PI), Christian Medical College, Vellore, India

Miklos Barta, Consultant Radiologist, Royal Cornwall Hospital, Treliske, UK

Indrani Bhattacharya, Consultant Clinical Oncologist (PI), Cambridge University Hospitals NHS Foundation Trust, Cambridge, UK

Matthew Blackledge, Team Leader, Radiotherapy & Imaging, The Institute of Cancer Research, London, UK

Philip Borg, Consultant Radiologist, The Christie NHS Foundation Trust, Manchester, UK

Fay Cafferty, Principal Statistician, The Institute of Cancer Research Clinical Trials and Statistics Unit (ICR-CTSU), The Institute of Cancer Research, London, UK

Fiona Castell, Consultant Clinical Oncologist, King’s College Hospital NHS Foundation Trust, London, UK

Rakesh C.A., Consultant Radiologist, Regional Cancer Centre, Trivandrum, India

Aditi Chandra, Consultant Radiologist, Tata Medical Centre, Kolkata, India

Sanjoy Chatterjee, Consultant Clinical Oncologist (PI), Tata Medical Centre, Kolkata, India

Daljit Gahir, Consultant Clinical Oncologist (PI), University Hospitals of North Midlands, Stoke on Trent, UK

Lone Gothard, Senior Research Coordinator, The Institute of Cancer Research, London, UK

Purvi Haria, Consultant Radiologist, Tata Memorial Centre, Mumbai, India

Nuala Healy, Consultant Radiologist, Cambridge University Hospitals NHS Foundation Trust, Cambridge, UK

Anna Kirby, Consultant Clinical Oncologist, The Royal Marsden NHS Foundation Trust, Sutton, UK

Lorna Leonard, Commercial Study Oversight Manager, The Institute of Cancer Research Clinical Trials and Statistics Unit (ICR-CTSU), The Institute of Cancer Research, London, UK

Dayananda Lingegowda, Consultant Radiologist, Tata Medical Centre, Kolkata, India

Imogen Locke, Consultant Clinical Oncologist, The Royal Marsden NHS Foundation Trust, Sutton, UK

Mairead MacKenzie, Patient Representative, Independent Cancer Patients’ Voice, London, UK

Beela Mathew, Professor, Radiation Oncology (PI), Regional Cancer Centre, Trivandrum, India

Alison Ranger, Consultant Clinical Oncologist, The Royal Marsden NHS Foundation Trust, London, UK

Shalini Sahu, Consultant Radiologist, Christian Medical College, Vellore, India

Seema Salehi-Bird, Consultant Breast Radiologist, University Hospitals of North Midlands, Stoke on Trent, UK

Elinor Sawyer, Consultant Clinical Oncologist, Guy’s and St Thomas’ NHS Foundation Trust, London, UK

Archana Seth, Consultant Radiologist, Beatson Oncology Centre, Glasgow, UK

Victoria Sinnett, Consultant Radiographer, The Royal Marsden NHS Foundation Trust, Sutton, UK

Alastair Thomson, Consultant Clinical Oncologist (PI), Royal Cornwall Hospitals NHS Trust, Truro, UK

Karen Venables, RTTQA Group Lead, Mount Vernon Hospital, Northwood, UK

Tabassum Wadasadawala, Professor and Radiation Oncologist (PI), Tata Memorial Center, Mumbai, India

Charlotte Westbury, Consultant Clinical Oncologist, East and North Hertfordshire NHS Trust, Northwood, UK

Virginia Wolstenholme, Consultant Clinical Oncologist, Barts Health NHS Trust, London, UK

John Yarnold, Professor of Clinical Oncology, The Institute of Cancer Research, London, UK
